# Difficulty in differentiating liver injury from an immune checkpoint inhibitor from chemotherapy

**DOI:** 10.3389/fphar.2024.1453595

**Published:** 2024-08-16

**Authors:** Shike Lou, Xiaoyin Wang, Fei Yuan, Gangde Zhao, Mingyang Feng, Yezhou Ding, Lanyi Lin, Kehui Liu, Xiaolin Wang, Wanqing Chi, Hui Wang

**Affiliations:** ^1^ Department of Infectious Diseases, Ruijin Hospital, Shanghai Jiao Tong University School of Medicine, Shanghai, China; ^2^ Department of Pathology, Ruijin Hospital, Shanghai Jiao Tong University School of Medicine, Shanghai, China; ^3^ Epidemiology of Microbial Disease, Yale University School of Public Health, New Haven, CT, United States

**Keywords:** immune checkpoint inhibitors (ICIs), chemotherapy, DILI, combined therapy, lung adenocarcinoma, liver biopsy

## Abstract

This study investigated the potential of immune checkpoint inhibitors (ICIs) combined with chemotherapy as a promising treatment approach for malignancies. This report focuses on a patient with drug-induced liver injury (DILI) following the administration of chemotherapy and ICIs. A 63-year-old patient with non-small cell lung adenocarcinoma (NSCLC) initially underwent γ-knife treatment and subsequently received a combination of chemotherapy comprising bevacizumab and camrelizumab. Due to liver abnormalities, both chemotherapy and ICIs were stopped on day 21. The patient’s liver function improved within a month after methylprednisolone treatment. Subsequently, the patient received carboplatin, pemetrexed, and bevacizumab without complications. This finding supported the notion that DILI was likely triggered by the ICI. This case series details a complex instance of DILI resulting from the use of ICIs and pemetrexed/carboplatin. The alignment of the pathological findings and clinical presentation strongly suggested ICI-induced DILI, which was further supported by the definitive response to steroid treatment. This information is important for clinicians, as it emphasizes the importance of closely monitoring liver function and being aware of potential adverse effects associated with ICIs. Such insights contribute to more effective patient care.

## Introduction

Immune checkpoint inhibitors (ICIs) are currently being extensively utilized in the treatment of malignancies and have yielded promising outcomes (Benitez et al., 2020). ICIs are at the forefront of pharmacological intervention for non-small cell lung carcinoma (NSCLC) treatment (Santoni et al., 2023) owing to the promising potential of immunotherapy for achieving complete remission. The effectiveness of ICIs, however, could be influenced by various factors, including acidic microenvironments (Rizzo et al., 2022) and/or gender (Santoni et al., 2022). In addition, the effectiveness of ICIs is sometimes compromised because of the occurrence of severe immune-related adverse events (irAEs) (Sosa et al., 2018), such as those involving the skin, gastrointestinal system, thyroid, and liver. Among these irAEs, liver injury is one of the most common types of injury, accounting for nearly 16% of cancer patients receiving ICI treatment (Peeraphatdit et al., 2020). Furthermore, drug-induced liver injury (DILI) can be induced by various agents used against cancer (Bahirwani and Reddy, 2014), including ICIs (Zen and Yeh, 2018) and chemotherapy (Bahirwani and Reddy, 2014). However, ICI-induced DILI is strongly correlated with obesity in patients (Lou et al., 2023), supporting findings concerning sex hormone levels (Santoni et al., 2022) because obesity has the potential to disrupt proper functioning of the endocrine system. Furthermore, DILI can also be induced by cancer chemotherapeutic agents. In the present report, we describe a case in which a patient with NSCLC experienced DILI while receiving a concurrent regimen of chemotherapy and ICIs. However, it remains unclear whether the development of DILI was a consequence of ICIs and/or combined chemotherapy and ICI treatment, complicating diagnosis and subsequent management.

## Case

A 63-year-old male patient with NSCLC exhibited neurological dysfunction stemming from NSCLC metastasis to the central nervous system, showing slurred speech and decreased memory.

**FIGURE 1:**
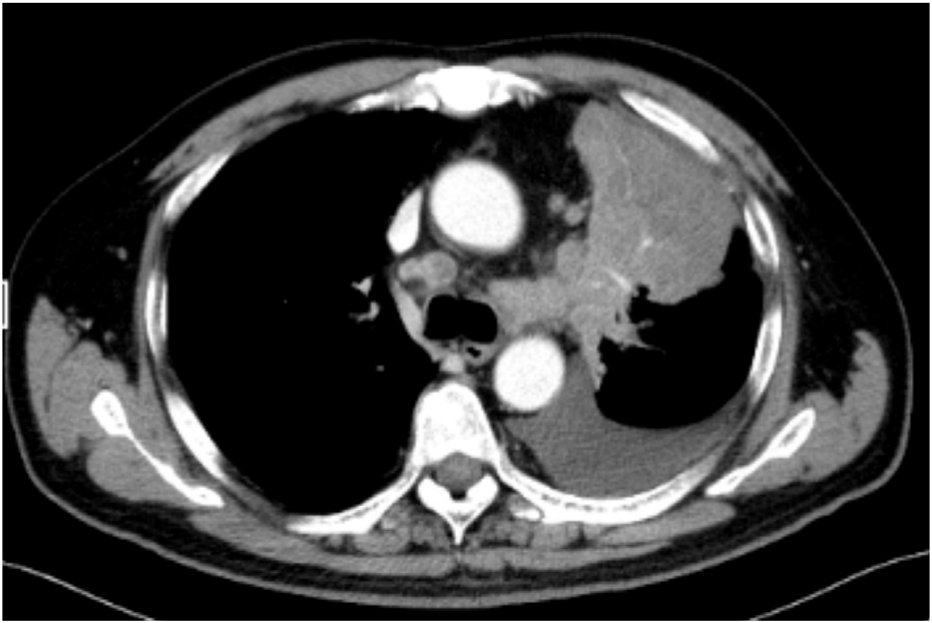
Enhanced CT scan of the chest.

Contrast-enhanced chest computed tomography (CT) confirmed primary cancer in the upper lobe of the left lung, with a maximum length of 7.8 cm × 7.5 cm (Figure 1). MRI demonstrated metastatic lesions in the brain (pons, bilateral cerebellum, bilateral frontal and parietal lobes; multiple lesions in the left temporal lobe with peritumoral oedema); and multiple metastatic lesions in the liver.

Histopathological analysis of the pleural effusion drainage fluid was performed, confirming the presence of NSCLC. Additionally, immunohistochemistry (IHC) of the cells from the drainage fluid revealed a 20% positive expression rate of PD-L1.

Pathology of the NSCLC specimen revealed that the TNM stage was T4, Nx, or M1c, and the differentiation was IVB. In addition, a 20% PD-L1 tumour proportion was coupled with an Exon 2 KRAS (Kirsten rat sarcoma viral oncogene homolog) mutation. No EGFR (epidermal growth factor receptor) or ALK (anaplastic lymphoma kinase) rearrangements were detected.

Chemotherapy and bevacizumab were initiated 23 days after second γ-knife treatment. The patient had camrelizumab on the next day of chemotherapy, and underwent just one round of such ICI and chemotherapy within 2 weeks. On day 1, the treatment plan included chemotherapy, involving carboplatin (550 mg) and pemetrexed (1,000 mg), along with the administration of bevacizumab [a monoclonal antibody targeting VEGF (vascular endothelial growth factor)] (600 mg) and camrelizumab (an anti-PD-1 monoclonal antibody) (200 mg) on day 2. The patient developed a maculopapular rash on the chest accompanied by itching, as well as tongue ulcers, during the course of treatment on day 3. Fortunately, these symptoms spontaneously resolved by day 6.

**FIGURE 2:**
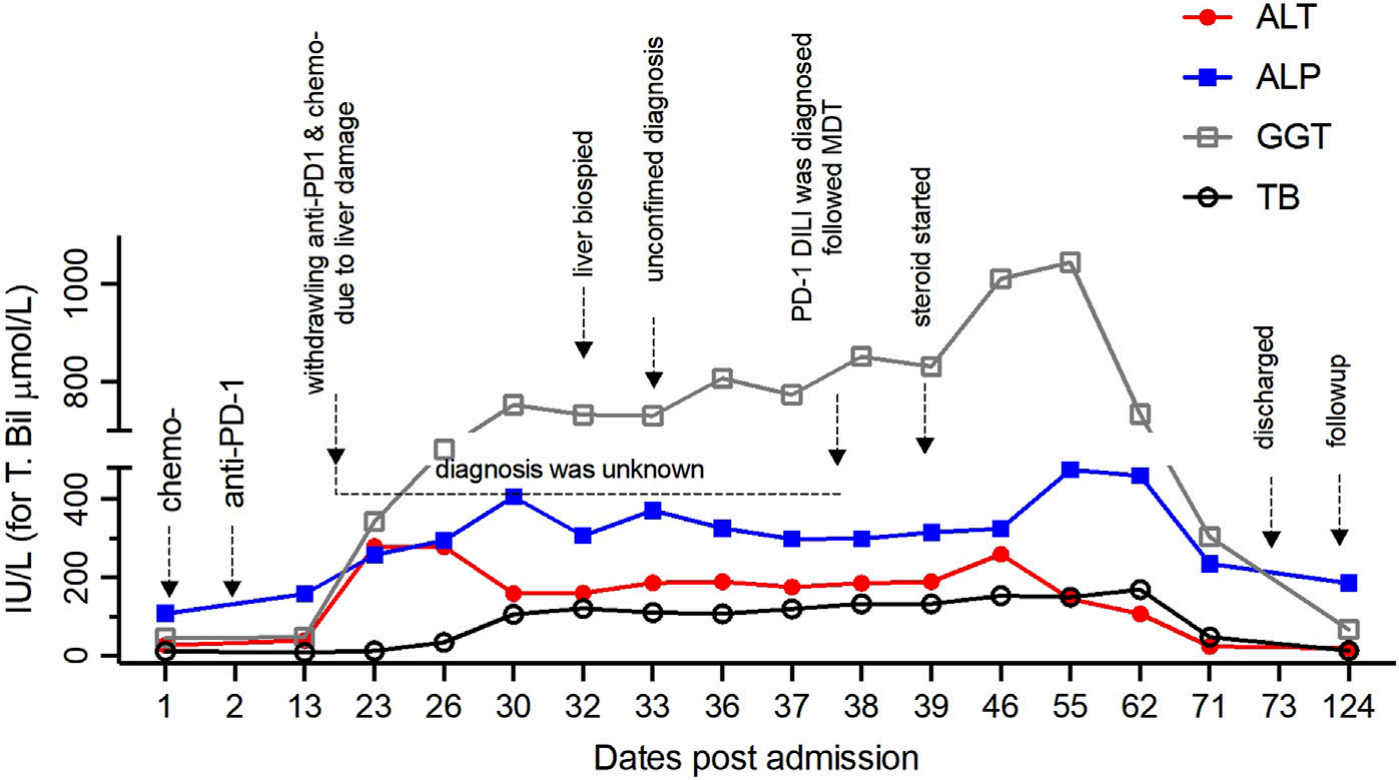
The kinetics of biochemical parameters reflecting liver function before and after withdrawal of ICIs and chemotherapy over the entire period are shown, with indicators for every approach.

On day 13, however, abnormal liver function was identified, characterized by elevated alanine transaminase (ALT), aspartate transaminase (AST), alkaline phosphatase (ALP), and gamma-glutamyl transferase (GGT), measuring 268 IU/L, 280 IU/L, 259 IU/L, and 344 IU/L, respectively. Total and direct bilirubin levels were initially within the normal range at 12.7 and 3.9 μmol/L, respectively (Figure 2). An investigation was initiated to determine the cause of the abnormal liver function. Viral hepatitis, autoimmune liver disease, and space-occupying lesions were ruled out because no viral presence or autoantibodies were detected and because the liver and bile duct structure appeared normal. However, the specific cause of this liver injury has remained unclear. If DILI was the underlying cause, it was unclear which specific medication(s) was responsible. Consequently, both the ICIs and chemotherapy were discontinued.

**FIGURE 3:**
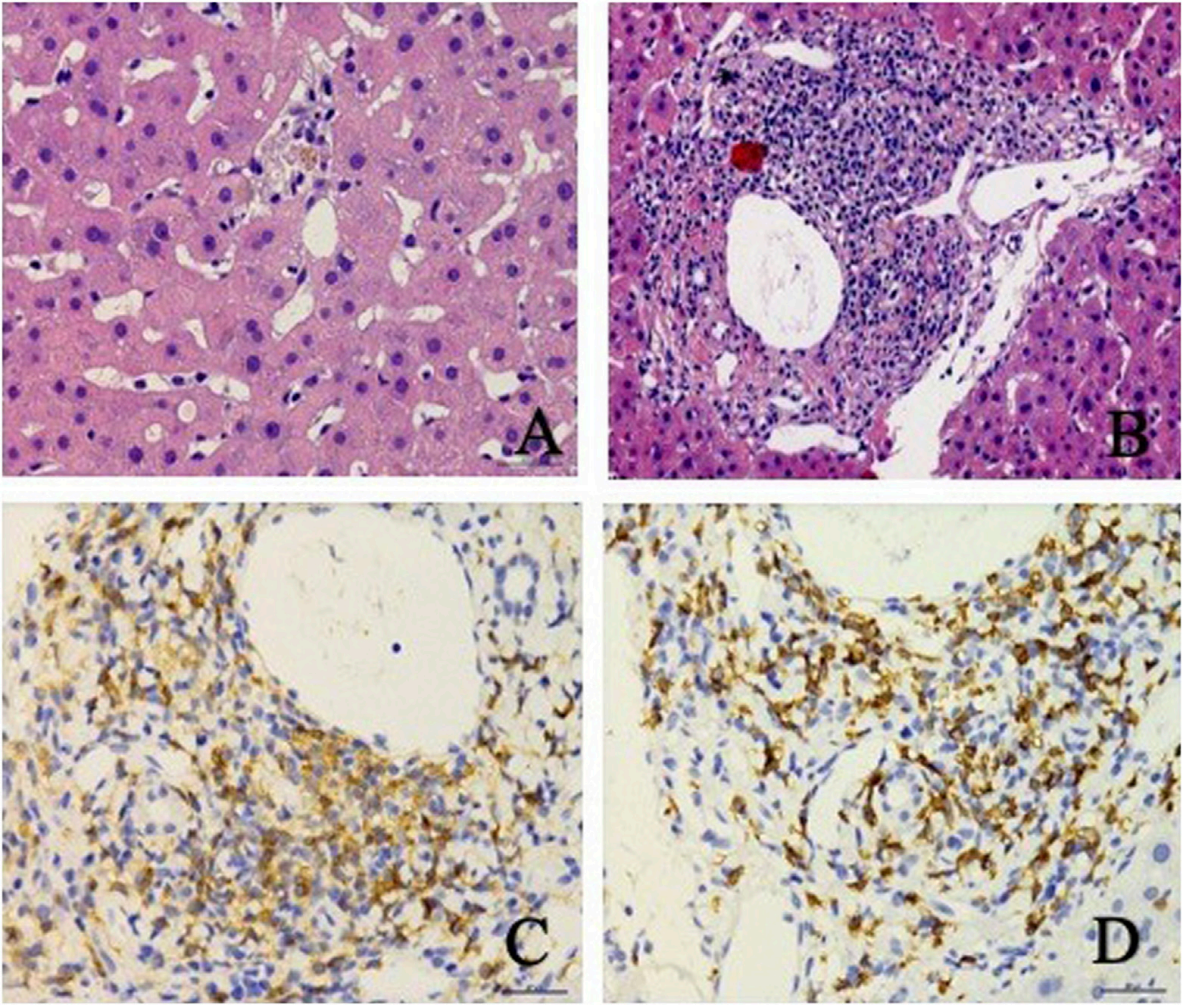
Liver biopsy revealed patchy focal necrosis **(A)**, and infiltrating inflammatory cells, mainly lymphocytes in the portal area **(B)**, and enrichment of CD4^+^ **(C)** and CD8^+^ **(D)** in the portal area.

On day 32, histopathological examination of the liver biopsy sample revealed the presence of focal necrosis, acidophilic bodies, mild steatosis and cholestasis (Figure 3). A lymphocytic infiltration was noted within the sinusoids and was found to be abundant within the portal area (Figures 3A,B). Despite these findings, the fundamental histopathology could not definitively establish a diagnosis. Subsequent immunohistochemical analysis revealed that the majority of infiltrating leukocytes were CD4^+^ and CD8^+^ lymphocytes that were equally abundant in the portal area, while CD20^+^ B lymphocytes were notably absent. No noticeable damage to the bile duct was evident following CK19 immunostaining. Furthermore, tests for hepatitis B surface antigen (HBsAg), hepatitis B core antigen (HbcAg), copper, and Pearls blue detection returned negative results. Notably, a genetic variant that may have an association with DILI, RS72631567, was found in this patient (Nicoletti et al., 2017). The RS72631567 variant has a RUCAM (Roussel Uclaf Causality Assessment Method) score at 6 (probable association).

Notwithstanding these supplementary investigations, a conclusive diagnosis has remained elusive; nonetheless, a suspicion of ICI-induced DILI persisted based on the observed histopathology and immunohistochemistry results.

Consequently, on day 37, a multidisciplinary team (MDT) involving oncologists, radiologists, pathologists, hepatologists, and pharmacologists was convened. According to the MDT, the most plausible explanation for liver injury was ICI-induced immune-related hepatotoxicity, which was categorized at level 3 according to the Common Adverse Reaction Toxicity Criteria (CTCAE 5.0, 2017 version) from the National Cancer Institute (US National Cancer Institute, 2017).

On day 38, given the intricacy of this case, a second pathological consultation was sought from an external specialized hepatology unit. This consultation strongly substantiated the diagnosis of ICI-induced DILI. On day 39, diagnostic treatment involving the administration of methylprednisolone was initiated, starting from intravenous injection of methylprednisolone 40 mg twice a day for 3 days. The dose of methylprednisolone was increased to 60 mg twice a day for 4 days due to poor alleviation of liver injury. The dose of intravenous methylprednisolone was reduced to 40 mg twice a day due to the decline of liver enzymes. After 2 weeks of intravenous maintenance, the patient’s liver function further improved. Intravenous methylprednisolone was replaced by prednisone 10 mg once a day orally. The dose was reduced to prednisone 5 mg once a day orally 11 days later. Glucocorticoids therapy lasts 42 days in total. On day 71, his liver function had almost fully recovered, evident through the nearly normalized levels of ALT, AST, ALP, and GGT by the time of discharge (Figure 2).

On day 124 after discharge, the patient tolerated a subsequent round of chemotherapy (consisting of carboplatin and pemetrexed) combined with administration of bevacizumab, excluding the use of ICIs. Remarkably, this course of treatment was devoid of any adverse events.

## Discussion

The primary manifestation of ICI-induced DILI involves an abnormal increase in AST and ALT levels (De Martin et al., 2018). A cholestatic hepatitis pattern of injury is observed in more than 50% of cases (Mizuno et al., 2020), followed by a mixed liver injury pattern, while hepatocellular liver injury represents the least common pattern (Imoto et al., 2019).

In the present case, the patient developed DILI following the administration of a combination of pemetrexed and carboplatin (chemotherapy) along with camrelizumab (ICIs). Consequently, pinpointing the exact cause of this DILI proved to be quite challenging. However, the patient’s presentation, characterized by precordial pruritic maculopapular rash and tongue ulceration, aligned more closely with ICI-induced liver injury. This finding aligns consistently with the observed abnormal liver function and the pathological findings from the liver biopsy.

We should consider that the condition of this patient was ICI-induced DILI during the treatment of NSCLC because of the ongoing debate surrounding whether ICIs represent the optimal choice for initial drug therapy in NSCLC (Rizzo, 2022). Chemotherapy, as a potential cause of DILI, typically correlates with more severe histopathological hepatic damage, involving both hepatic necrosis and damage to the epithelial cells lining the bile ducts (Zen et al., 2020). In contrast, our examination revealed an abundance of CD3^+^/CD8^+^ lymphocytes, hepatocyte patch necrosis, and an inflammatory response within the portal area without evident damage to the hepatic or bile ducts. Thus, the histological and immunohistochemical characteristics were more consistent with ICIs being the likely culprits for DILI in these patients (De Martin et al., 2018).

While the MDT assessment did not yield a definitive conclusion based on the amalgamation of clinical presentation and pathological findings, the diagnosis leaned toward ICIs as the probable cause of DILI in this patient. This finding aligns well with previously published data on ICI-induced DILI (Zen et al., 2020), which indicate that infiltrating lymphocytes in the portal vein region consist of CD4^+^ helper T cells and/or CD8^+^ cytotoxic T cells, while those within the lobules are predominantly CD8^+^ cytotoxic T cells (De Martin et al., 2018).

Subsequently, diagnostic treatment with methylprednisolone confirmed the causative link, with liver function showing almost complete recovery within 1 month. The patient exhibited successful tolerance to subsequent carboplatin, pemetrexed, and bevacizumab therapy, further reinforcing the hypothesis that the DILI in this patient was indeed attributed to ICI exposure.

There are several limitations of the current study. First, the initiation of steroids was substantially delayed by 4 weeks, and the withdrawal of ICIs and chemotherapy was stopped after 2 weeks due to inexperience with such scenarios at the time of ICI therapy and/or chemotherapy-induced DILI. This case emphasizes the significance of recognizing ICI-related hepatitis as a potential form of DILI in patients who exhibit elevated ALT levels indicative of grade 4 ICI-related hepatitis risk, in line with the 2022 National Comprehensive Cancer Network (NCCN) guidelines (Thompson et al., 2022). The guidelines propose prompt steroid therapy initiation along with temporary discontinuation of ICI therapy for such patients.

Second, this led to challenges in pinpointing and confirming the agent(s) responsible for the DILI, whether it was the ICI, the chemotherapy, or their combined application. Nonetheless, the comprehensive documentation of this unique case presented here provides valuable insights for clinicians managing cancer patients, particularly when confronted with adverse reactions stemming from concurrent ICI and chemotherapy usage.

Furthermore, the lack of experience in managing such patients at that time led to our failure to recognize that the chemical agents used (carboplatin and pemetrexed) generally posed a low to moderate risk of transaminitis, typically without causing substantial liver injury or jaundice (Pemetrexed and carboplatin, 2024). This case provides valuable insight into the potential hepatotoxicity induced by ICIs, offering instructive information for fellow clinicians.

Although this patient was diagnosed with ICI-induced DILI, it remains unexplored whether there was a synergistic effect between ICIs and other chemotherapeutic agents. This synergy might induce significant necrosis/apoptosis of malignant cells and further escalate inflammation within the microenvironment, potentially complicating disease progression compared to the normal course (Bahirwani and Reddy, 2014). Unfortunately, we cannot exclude the possibility of synergistic liver injury resulting from the combined administration of ICIs and chemotherapy, primarily due to the impracticality of establishing a suitable control group for both ethical and technical reasons.

To conclude, we present a complex case involving DILI following the administration of ICIs. The alignment of pathological findings with clinical presentation strongly suggested ICI-induced DILI, a hypothesis further substantiated by the subsequent conclusive diagnostic response to steroids. However, we can’t rule out if the patient could recover smoothly without methylprednisolone. This case study provides crucial insights for clinicians, underscoring the necessity of vigilance concerning the potential adverse effects of ICIs and highlighting the importance of a more effective patient management approach, with particular emphasis on meticulous monitoring of liver function.

## Data Availability

The raw data supporting the conclusions of this article will be made available by the authors, without undue reservation.
